# Virtual Care and Electronic Patient Communication During COVID-19: Cross-sectional Study of Inequities Across a Canadian Tertiary Cancer Center

**DOI:** 10.2196/39728

**Published:** 2022-11-04

**Authors:** Amir H Safavi, Mike Lovas, Zhihui Amy Liu, Sheena Melwani, Tran Truong, Shayla Devonish, Nazek Abdelmutti, Ambreen Sayani, Danielle Rodin, Alejandro Berlin

**Affiliations:** 1 Radiation Medicine Program Princess Margaret Cancer Centre Toronto, ON Canada; 2 Department of Radiation Oncology University of Toronto Toronto, ON Canada; 3 Smart Cancer Care Program Princess Margaret Cancer Centre Toronto, ON Canada; 4 Healthcare Human Factors University Health Network Toronto, ON Canada; 5 Princess Margaret Cancer Centre University Health Network Toronto, ON Canada; 6 Dalla Lana School of Public Health University of Toronto Toronto, ON Canada; 7 Data Science Program Princess Margaret Cancer Centre Toronto, ON Canada; 8 Techna Institute University Health Network Toronto, ON Canada; 9 Women's College Research Institute Women's College Hospital Toronto, ON Canada

**Keywords:** digital health, telehealth, telemedicine, eHealth, oncology, cancer care, virtual care, health inequities, health inequality, digital divide, COVID-19, electronic mail, cross sectional, engagement, satisfaction, patient reported, experience

## Abstract

**Background:**

Virtual care (VC) visits (telephone or video) and email-based patient communication have been rapidly adopted to facilitate cancer care during the COVID-19 pandemic. Inequities in access and patient experience may arise as these digital health tools become prevalent.

**Objective:**

We aimed to characterize inequities in access and patient-reported experience following adoption of digital health tools at a tertiary cancer center during the COVID-19 pandemic.

**Methods:**

We designed a cross-sectional study of outpatients with visits from September to December 2020. Patient characteristics and responses to an email-based patient-experience survey were collated. Inequities in access were assessed across three pairs of comparison groups: (1) patients with VC and in-person visits, (2) patients with and without documented email addresses, and (3) responders and nonresponders to the survey. Inequities in patient-reported experience were assessed among survey responders. Demographics were mapped to area-level averages from national census data. Socioeconomic status was mapped to area-level dimensions of the Canadian Index of Multiple Deprivation. Covariate balance between comparison groups was assessed using standardized mean differences (SMDs), with SMD≥0.2 indicating differences between groups. Associations between patient experience satisfaction scores and covariates were assessed using multivariable analyses, with *P*<.05 indicating statistical significance.

**Results:**

Among the 42,194 patients who had outpatient visits, 62.65% (n=26,435) had at least one VC visit and 31.15% (n=13,144) were emailable. Access to VC and email was similar across demographic and socioeconomic indices (SMD<0.2). Among emailable patients, 21.84% (2870/13,144) responded to the survey. Survey responsiveness was similar across indices, aside from a small difference by age (SMD=0.24). Among responders, 24.4% received VC and were similar to in-person responders across indices (SMD<0.2). VC and in-person responders had similar satisfaction levels with all care domains surveyed (all *P*>.05). Regardless of visit type, patients had variable satisfaction with care domains across demographic and socioeconomic indices. Patients with higher ethnocultural composition scores were less satisfied with the cultural appropriateness of their care (odds ratio [OR] 0.70, 95% CI 0.57-0.86). Patients with higher situational vulnerability scores were less satisfied with discussion of physical symptoms (OR 0.67, 95% CI 0.48-0.93). Patients with higher residential instability scores were less satisfied with discussion of both physical (OR 0.81, 95% CI 0.68-0.97) and emotional (OR 0.86, 95% CI 0.77-0.96) symptoms, and also with the duration of their visit (OR 0.85, 95% CI 0.74-0.98; *P*=.02). Male patients were more satisfied with how their health care provider had listened to them (OR 1.64, 95% CI 1.11-2.44; *P*=.01).

**Conclusions:**

Adoption of VC and email can equitably maintain access and patient-reported experience in cancer care across demographics and socioeconomic indices. Existing health inequities among structurally marginalized patients must continue to be addressed to improve their care experience.

## Introduction

Virtual care (VC), referring to the delivery of care using information and telecommunications technologies [1], has been adopted at cancer centers during the COVID-19 pandemic to promote adherence to physical distancing and other public health measures [2-5]. At the Princess Margaret Cancer Centre (Toronto, Ontario), VC visits were rapidly implemented 12 days after declaration of the pandemic to reduce in-person visits by 50% while maintaining continuity of care [2]. The hospital-wide VC platform developed in-house was also leveraged to email patient-reported experience surveys to all patients after VC or in-person visits, allowing quality improvement data collection to continue during the pandemic [2].

As digital health tools such as VC and email-based patient communications are increasingly adopted, it is possible they may mitigate or exacerbate existing health inequities [6]. Differential access to and benefit from digital resources is termed the digital divide [7-9]. Digital divides are modulated by the social determinants of health, including age, gender, income, housing, rurality, race, and language. Some have noted the potential benefits of VC and email for enhancing equitable care [10-12], including increased health care utilization among racialized minorities and those with travel restrictions. Others have expressed concerns about digital divides in access to VC due to structural marginalization [13-17], particularly among marginalized populations known to be more prone to adverse oncologic outcomes [18,19]. These concerns underscore the importance of investigating inequities following the adoption of digital health tools and addressing their impact on oncologic care [20,21]. Herein, we aimed to characterize inequities in access and patient-reported experience following adoption of digital health tools at a Canadian tertiary cancer center.

## Methods

### Study Design

A cross-sectional study was designed. Inequities in access were assessed across three pairs of comparison groups: (1) patients with one or more VC visits and those with only in-person visits; (2) emailable and nonemailable patients (effectively, patients with and without documented email addresses); and (3) responders and nonresponders to an email-based, patient-reported experience survey. Inequities in patient-reported experience were assessed among survey responders. We characterized digital divides in access to and use of VC and email using group comparisons by demographics and clinic type. We characterized inequities in patient experience among all patients by identifying associations between satisfaction with care and demographics or visit type. This study was conducted in accordance with the STROBE (Strengthening the Reporting of Observational Studies in Epidemiology) reporting guidelines.

### Setting and Participants

All patients with outpatient VC or in-person clinic visits at the Princess Margaret Cancer Centre, University Health Network (Toronto, Ontario, Canada) from September 1 through December 31, 2020, were included in this study. This center conducts approximately 2000 outpatient visits daily, including close to 1000 ambulatory clinic encounters and 1000 ambulatory procedures and treatments [2]. We extracted study data, including age, gender, postal codes, clinic type, and visit type (in-person vs VC), and completed patient-reported experience surveys from the electronic medical record system and the hospital-wide VC platform.

### Characterization of Demographics

The following demographics were selected for use in this study: age, gender, income, low-income status, area type, and the four dimensions of the Canadian Index of Multiple Deprivation (CIMD), namely residential instability (RI), economic dependency (ED), ethnocultural composition (EC), and situational vulnerability (SV) (Multimedia Appendix 1). Age and gender data were extracted from the electronic medical record, while the other demographics were derived from dissemination area (DA)-level data reported in the Statistics Canada 2016 census. Each individual’s DA was captured by linking their postal code to Statistics Canada 2016 census data using Postal Code Conversion File (PCCF+) version 7B.

Individual patient income was estimated using the neighborhood income per single-person equivalent, a household size–adjusted measure of household income (before tax), based on 2016 Census Summary profile data at the DA level. Low-income status was determined by comparing neighborhood income per single-person equivalent to Statistics Canada low-income cutoffs in 2016, based on area type (rural vs small vs medium vs large population center) and family size of one person. Per Statistics Canada, rural was defined as a population less than 1000, small population was defined as 1-29,999, medium population was defined as 30-99,999, and large population was defined as 100,000 or more. Socioeconomic status was mapped to the four DA-level dimensions of the CIMD (RI, ED, EC, and SV). For each dimension, we provided the CIMD in two forms: factor scores (higher scores correspond to more marginalized areas) and quintiles (a value of 1 corresponds to the least deprived area and 5 corresponds to the most deprived area). The constituent elements of each CIMD dimension are significantly correlated with only one dimension and are described in Multimedia Appendix 1.

### Patient-Reported Experience Survey

We emailed an adapted version of Your Voice Matters [22], a validated patient-reported experience survey provincially mandated by Ontario Health, to all patients with documented email addresses at the Princess Margaret Cancer Centre after each outpatient VC or in-person clinic visit starting in September 2020. The survey was available in eight languages: English, French, Simplified Chinese, Traditional Chinese, Spanish, Portuguese, Italian, and Vietnamese. The adapted survey included new questions regarding the utilization of VC [23] (Multimedia Appendix 1, section 1.2; Q3-5), as well as existing questions regarding satisfaction with various care domains such as discussion of physical and emotional symptoms and cultural appropriateness of care. The survey is found in Multimedia Appendix 1 (section 1.2).

### Statistical Methods

Summary statistics were calculated to describe demographics and clinic types of the full cohort and comparison groups. Categorical variables are summarized as numbers (percentages) and continuous variables are summarized as means (SD), medians (IQR), deciles (for income), and quintiles (for RI, ED, EC, and SV). The first completed survey from each respondent was utilized for this analysis. A sensitivity analysis was performed to assess the influence of intrapatient correlation among all survey responses (if there were multiple responses from the same respondent) on the overall results. Within survey responders, a large proportion reported their satisfaction as 4 or 5 out of 5, and therefore responses were dichotomized for each question as “satisfied” (reported 4 or 5) and “not satisfied” (reported 1, 2, or 3). Standardized mean differences (SMDs) were calculated for group comparisons by demographic variable and clinic types; SMDs of 0.2, 0.5, and 0.8 were considered small, medium, and large differences, respectively. Multivariable logistic regression was used to assess associations between satisfaction scores and demographic variables, as well as visit type, with income on a log scale. Statistical significance was judged at *P*<.05. Complete case analyses were performed to address missing survey responses. To correct for bias due to nonresponse, multivariable models for outcome variables were fitted with potential predictors of nonresponse as covariates [24]. Analyses were performed using R version 4.0.2 (R Foundation for Statistical Computing).

### Ethics Statement

This study was reviewed by the institutional research ethics board; ethics approval was obtained, along with a waiver for written informed consent (University Health Network Quality Improvement Review Committee #21-0148).

## Results

### Participant Characteristics

From September 2020 through December 2020, 42,194 patients had outpatient clinic visits (Table 1). The median age of the full cohort was 64 (IQR 52-73) years and 51.7% of the patients self-identified as male. The majority of patients lived in a large urban population center. The mean income of the full cohort was CAD $62,400 (SD CAD $27,700), with CAD $1=~US $0.75. Approximately 7% of patients were classified in a low-income category. For the full cohort, the most common clinical specialties visited were genitourinary, head and neck, and gastrointestinal oncology. Full cohort characteristics are listed in Table S1 of Multimedia Appendix 1.

**Table 1:** Patient demographic characteristics of the full cohort (N=42,194).

Characteristics		Value
**Age (years)**		
	Mean (SD)	61.6 (15.5)
	Median (IQR)	64 (52 to 73)
**Gender, n (%)**		
	Female	20,388 (48.32)
	Male	21,806 (51.68)
**Area^a^, n (%)**		
	Rural area	2830 (6.71)
	Small population center	2127 (5.04)
	Medium population center	1494 (3.54)
	Large urban population center	35,743 (84.71)
**BTIPPE^b^ (CAD $^c^)×1000**		
	Mean (SD)	62.4 (27.7)
	Median (IQR)	58.4 (46.2 to 72.6)
	1st decile, n (%)	3594 (8.52)
	2nd decile, n (%)	3668 (8.69)
	3rd decile, n (%)	3888 (9.21)
	4th decile, n (%)	3925 (9.30)
	5th decile, n (%)	3795 (8.99)
	6th decile, n (%)	3899 (9.24)
	7th decile, n (%)	3750 (8.89)
	8th decile, n (%)	4103 (9.72)
	9th decile, n (%)	4822 (11.43)
	10th decile, n (%)	6750 (16.00)
**Low income^d^, n (%)**		
	No	39,173 (92.84)
	Yes	3021 (7.16)
**Residential instability^e^**		
	Score, mean (SD)	0.3 (1.2)
	Score, median (IQR)	–0.1 (–0.6 to 1.2)
	1st quintile, n (%)	6747 (15.99)
	2nd quintile, n (%)	7025 (16.65)
	3rd quintile, n (%)	7100 (16.83)
	4th quintile, n (%)	7500 (17.78)
	5th quintile, n (%)	13,822 (32.76)
**Economic dependency^e^**		
	Score, mean (SD)	–0.1 (1.1)
	Score, median (IQR)	–0.2 (–0.8 to 0.4)
	1st quintile, n (%)	10,764 (25.51)
	2nd quintile, n (%)	8685 (20.58)
	3rd quintile, n (%)	7983 (18.92)
	4th quintile, n (%)	7398 (17.53)
	5th quintile, n (%)	7364 (17.45)
**Ethnocultural composition^e^**		
	Score, mean (SD)	0.5 (1.1)
	Score, median (IQR)	0.3 (–0.4 to 1.2)
	1st quintile, n (%)	2663 (6.31)
	2nd quintile, n (%)	4616 (10.94)
	3rd quintile, n (%)	7606 (18.03)
	4th quintile, n (%)	12,195 (28.90)
	5th quintile, n (%)	15,114 (35.82)
**Situational vulnerability^e^**		
	Score, mean (SD)	–0.3 (0.8)
	Score, median (IQR)	–0.5 (–0.8 to 0.1)
	1st quintile, n (%)	14,348 (34.00)
	2nd quintile, n (%)	9219 (21.85)
	3rd quintile, n (%)	7277 (17.25)
	4th quintile, n (%)	6126 (14.52)
	5th quintile, n (%)	5224 (12.38)
**Clinic type, n (%)**		
	Genitourinary	8885 (21.06)
	Head and neck	4594 (10.89)
	Gastrointestinal	4220 (10.00)
	Breast	3478 (8.24)
	Gynecologic	3316 (7.86)
	Other	17,701 (41.95)

^a^Rural area was defined as a population of less than 1000, small population was defined as 1-29,999 people, medium population was defined as 30-99,999 people, and large population was defined as 100,000 or more people.

^b^BTIPPE: before-tax neighborhood income per single-person equivalent.

^c^CAD $1=~US $0.75.

^d^Low-income status refers to neighborhood income per single-person equivalent below the Statistics Canada low-income cutoffs in 2016, based on area type (rural vs small vs medium vs large population center) and family size of one person.

^e^Higher factor scores and quintiles correspond to more marginalized areas.

Among the full cohort (N=42,194), 26,435 patients (62.65%) had at least one VC visit and 13,144 patients (31.15%) were emailable (Table 2). Among the emailable patients, 2870 (21.84%) responded to the survey. The majority of patients (97%) completed the survey once; the first or only completed surveys were included in the subsequent analysis. Sensitivity analysis accounting for multiple completed surveys by the minority of patients (3%) did not identify undue influence from intrapatient correlation. The percentage of missing responses was low (<5%) for most survey questions (Tables S6-S10 and S14 of Multimedia Appendix 1). Almost all responders (97.9%) completed the survey in English (Table S5 of Multimedia Appendix 1). Among responders, 36.1% reported that they were explicitly provided the option to have an in-person or VC visit (Table S9 of Multimedia Appendix 1).

In-person and VC visits were reported by 73.2% and 24.4% of respondents, respectively (Table 3). A visit type was not reported by 2.4% of responders. Among VC respondents, 84.6% reported having a phone visit, while 15.4% reported having a video visit (Table S9 of Multimedia Appendix 1). Additional group characteristics are found in Table 2, Table 3, and Multimedia Appendix 1 (section 1.3).

**Table 2:** Effect size measurements of differences by visit type, access to email, and survey responsiveness.

Characteristics			Visit type							Access to email							Survey responsiveness				
			In-person only (n=15,759)		≥1 virtual visit (n=26,435)		SMD^a^		Nonemailable (n=29,050)			Emailable (n=13,144)		SMD		Nonresponder (n=10,274)			Responder (n=2870)		SMD
**Age**																					
	Mean (SD)	60.9 (15.4)		62 (15.5)		0.072		61.9 (15.6)			60.9 (15.4)		0.063		60.1 (15.8)			63.7 (13.4)		0.241	
	Median (IQR)	63.0 (52.0-72.0)		64.0 (53.0-73.0)		—^b^		64 (53-73)			63 (52-72)		—		62 (51-71)			65 (56-73)		—	
**Gender, n (%)**																					
	Female	8353 (53)		12,035 (45)		0.15		13,798 (47)			6590 (50)		0.053		5077 (49)			1513 (53)		0.066	
	Male	7406 (47)		14,400 (55)		—		15,252 (53)			6554 (50)		—		5197 (51)			1357 (47)		—	
Area type			—		—		0.097		—			—		0.047		—			—		0.008
**BTIPPE^c^ (CAD $^d^)×1000**																					
	Mean (SD)	60.9 (26.1)		63.3 (28.6)		0.09		62 (27.7)			63.3 (27.8)		0.045		62.5 (27.4)			66.3 (29)		0.137	
	Median (IQR)	57.6 (45.4-70.8)		58.8 (46.5- 73.4)		—		58.1 (45.8-72.1)			59 (46.8-73.4)		—		58.4 (46.3-72.6)			61.4 (49-76.5)		—	
	Decile distribution	—		—		0.088		—			—		0.068		—			—		0.182	
**Low income^e^, n (%)**																					
	No	14,816 (94)		24,357 (92)		0.074		26,993 (93)			12,180 (93)		0.01		9552 (93)			2628 (92)		0.053	
	Yes	943 (6)		2078 (8)		—		2057 (7)			964 (7)		—		722 (7)			242 (8)		—	
**Residential instability^f^**																					
	Score, mean (SD)	0.3 (1.2)		0.3 (1.2)		0.055		0.3 (1.2)			0.3 (1.2)		0.021		0.3 (1.2)			0.4 (1.2)		0.023	
	Quintile distribution	—		—		0.071		—			—		0.022		—			—		0.046	
**Economic dependency^f^**																					
	Score, mean (SD)	–0.1 (1.1)		–0.1 (1.1)		0.009		–0.1 (1.1)			–0.1 (1)		0.046		–0.1 (1)			–0.1 (1.1)		0.045	
	Quintile distribution	—		—		0.015		—			—		0.049		—			—		0.05	
**Ethnocultural composition^f^**																					
	Score, mean (SD)	0.5 (1.1)		0.5 (1)		0.012		0.5 (1.1)			0.5 (1)		0.006		0.5 (1.1)			0.3 (1)		0.186	
	Quintile distribution	—		—		0.081		—			—		0.057		—			—		0.177	
**Situational vulnerability^f^**																					
	Score, mean (SD)	–0.3 (0.8)		–0.3 (0.8)		0.069		–0.3 (0.8)			–0.4 (0.7)		0.078		–0.3 (0.8)			–0.4 (0.7)		0.156	
	Quintile distribution	—		—		0.07		—			—		0.082		—			—		0.15	
Clinic type			—		—		1.112		—			—		0.612		—			—		0.247

^a^SMD: standardized mean difference.

^b^Not applicable.

^c^BTIPPE: before-tax neighborhood income per single-person equivalent.

^d^CAD $1=~US $0.75.

^e^Low-income status refers to neighborhood income per single-person equivalent below the Statistics Canada low-income cutoffs in 2016, based on area type (rural vs small vs medium vs large population center) and family size of one person.

^f^Higher factor scores and quintiles for correspond to more marginalized areas.

**Table 3:** Effect size of differences by visit type among survey responders.

Characteristics		In-person visit (n=2101)	Virtual visit (n=700)	SMD^a^
**Age**				
	Mean (SD)	63.3 (13.7)	64.8 (13)	0.109
	Median (IQR)	65 (56-73)	67 (58-74)	—^b^
**Gender, n (%)**				
	Female	1163 (55.4)	319 (45.6)	0.197
	Male	938 (44.6)	381 (54.4)	—
Area type		—	—	0.093
**BTIPPE^c^ (CAD $^d^)×1000**				
	Mean (SD)	65.7 (28.3)	69.1 (30.9)	0.116
	Median (IQR)	61 (48.4-76.5)	64 (51.5-77.2)	—
	Decile distribution	—	—	0.176
**Low income^e^, n (%)**				
	No	1931 (91.9)	633 (90.4)	0.052
	Yes	170 (8.1)	67 (9.6)	
**Residential instability^f^**				
	Score, mean (SD)	0.4 (1.2)	0.3 (1.2)	0.06
	Quintile distribution	—	—	0.097
**Economic dependency^f^**				
	Score, mean (SD)	–0.1 (1.1)	–0.1 (1.1)	0.034
	Quintile distribution	—	—	0.1
**Ethnocultural composition^f^**				
	Score, mean (SD)	0.4 (1)	0.2 (0.9)	0.183
	Quintile distribution	—	—	0.18
**Situational vulnerability**				
	Score, mean (SD)	–0.4 (0.7)	–0.5 (0.7)	0.093
	Quintile distribution	—	—	0.109
Clinic type		—	—	0.646

^a^SMD: standardized mean difference.

^b^Not applicable.

^c^BTIPPE: before-tax neighborhood income per single-person equivalent.

^d^CAD $1=~US $0.75.

^e^Low-income status refers to neighborhood income per single-person equivalent below the Statistics Canada low-income cutoffs in 2016, based on area type (rural vs small vs medium vs large population center) and family size of one person.

^f^Higher factor scores and quintiles for correspond to more marginalized areas.

### Characterization of Digital Divides in Access to and Use of VC and Email

VC and in-person patients were similar across demographics, with negligible differences in age, gender, area, and income, as well as deprivation indices, including RI, ED, EC, and SV (Table 2). Clinic types differed between VC and in-person patients (SMD=1.112); the most common clinic types among VC patients were genitourinary (29.5%), gastrointestinal (11.0%), and breast (10.3%) oncology (Multimedia Appendix 1, Table S11). Emailable and nonemailable patients were similar across demographics (SMD<0.2). Clinic types differed between emailable and nonemailable patients (SMD=0.612); the most common clinic types among emailable patients were genitourinary (16.6%), gastrointestinal (11.0%), and breast (10.9%) oncology (Multimedia Appendix 1, Table S12). Survey responders and nonresponders were similar across demographics, aside from a small difference in age (SMD=0.241). Clinic types differed between responders and nonresponders (SMD=0.247); the most common clinic types among responders were genitourinary (15.1%), gynecologic (12.4%), and breast (12.2%) oncology (Multimedia Appendix 1, Table S13). VC and in-person respondents were also similar across demographics (SMD<0.2) (Table 3). Clinic types differed between VC and in-person respondents (SMD=0.646); the most common clinic types among VC respondents were genitourinary (29.1%), breast (11.9%), and lymphoma (9.4%) oncology (Multimedia Appendix 1, Table S14).

### Inequities in Patient Experiences Across Visit Types

**Figure 1:**
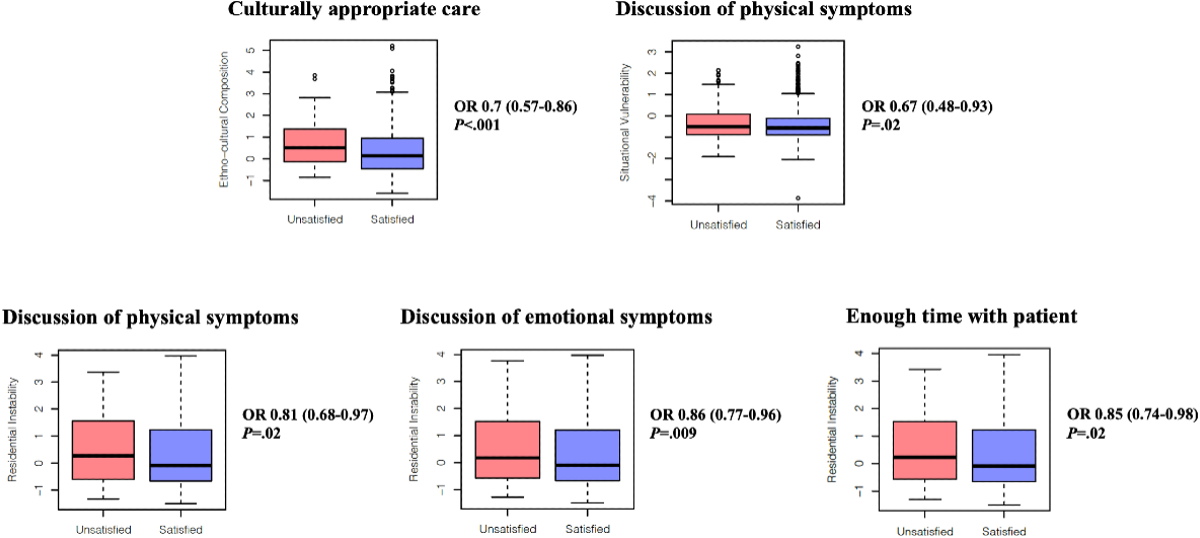
Associations between demographics and satisfaction with care quality domains. Values obtained from multivariable analyses of associations between demographics/visit type and satisfaction with surveyed care domains (see Multimedia Appendix 1 page 17-20). An odds ratio (OR)>1 for residential instability, ethnocultural composition, and situational vulnerability corresponds to increasing satisfaction with increasing marginalization. Box plots display the median value (central bar) and the IQR (lower and upper hinges denote the 75th and 25th percentiles, respectively). Whiskers indicate the minimum and maximum values within a distance of 1.5 times the IQR from the lower and upper hinges, respectively. Outlier data beyond the boundary of the whiskers are depicted by circles.

VC and in-person respondents had similar satisfaction levels with all care domains surveyed (all *P*>.05; Table 4), although VC respondents were less satisfied with their experience overall compared to in-person respondents (Table 4). Regardless of visit type, structurally marginalized patients were less satisfied with their care (Figure 1; Multimedia Appendix 1 Tables S15-S24). Patients with higher EC scores were less likely to characterize their care as culturally appropriate (odds ratio [OR] 0.7, 95% CI 0.57-0.89; *P*<.001). Patients with higher SV scores were less satisfied with discussion of physical symptoms (OR 0.67, 95% CI 0.48-0.93; *P*=.02). Patients with higher RI scores were less satisfied with discussion of physical (OR 0.81, 95% CI 0.68-0.97; *P*=.02) and emotional (OR 0.86, 95% CI 0.77-0.96; *P*=.009) symptoms. Patients with higher RI scores were also less satisfied that enough time had been spent with them during their visit (OR 0.85, 95% CI 0.74-0.98; *P*=.02). Male patients were more satisfied with how their health care provider had listened to them (OR 1.64, 95% CI 1.11-2.44; *P*=.01). Older patients were more satisfied with six of nine care domains surveyed.

**Table 4:** Associations between visit type (virtual vs in-person) and satisfaction with care quality domains.

Domain	OR^a^ (95% CI)^b^	*P* value
HCP^c^ listened to what you had to say?	0.83 (0.55-1.27)	.40
HCP discussed any of your physical symptoms?	0.93 (0.62-1.39)	.72
HCP discussed any of your emotional worries or concerns?	1.12 (0.87-1.45)	.37
HCP spent enough time with you?	1 (0.73-1.36)	.98
HCP let you ask questions?	0.88 (0.6-1.29)	.51
HCP explained things in a way you could easily understand?	1.07 (0.72-1.6)	.74
HCP involved you in decisions (choices) about your care in the way that you wanted?	1.09 (0.76-1.54)	.65
HCP provided care that you felt was appropriate given your ethnic/cultural background?	0.97 (0.62-1.51)	.88
HCP treated you with respect?	1.23 (0.69-2.2)	.48
Overall experience at your last visit?	0.68 (0.49-0.94)	.02

^a^OR: odds ratio.

^b^Values obtained from multivariable analyses of associations between demographics/visit type and satisfaction with surveyed care domains (see Multimedia Appendix 1 page 17-20); an odds ratio greater than 1 corresponds with greater satisfaction among patients with virtual care visits than those with in-person visits.

^c^HCP: health care provider.

## Discussion

### Principal Results

Our study quantitatively shows that VC and email are digital health tools that can equitably maintain patient access and experience at a publicly funded tertiary cancer center. Among the 42,194 patients analyzed, VC and email accessibility and survey responsiveness were similar across demographics and socioeconomic indices. These findings are encouraging, as continuing adoption of digital health tools in cancer care may improve clinical trial enrollment [20,25], patient-reported outcome collection [20,26], and timely participation in expert supportive care among underserved populations. Regardless of visit type, patients structurally marginalized by ethnocultural, situational, and residential status, as well as gender, were less satisfied with their care. These results reinforce the reality that social determinants of health have tangible impacts on the patient experience, and necessitate further characterization using targeted questionnaires and focus groups of patients, community members, and advocates, and engagement with these stakeholders in designing solutions for mitigating these inequities. These outcomes may also suggest that for the majority of patients, pre-existing social inequities, rather than the adoption of VC, may contribute to worse patient experiences. Thus, our work highlights the imperative for proactive and continuous quantification of social determinants of health to improve equitability of the patient experience.

### Comparison With Prior Work

Our work presents findings that may appear incongruent with other contemporary published studies. A recent analogous study based on administrative claims data from the United States found differential use of telemedicine by socioeconomic status among 16,006 newly diagnosed cancer patients during the COVID-19 pandemic [27]. Compared to our findings, the different conclusions in this study may be attributable to the differences between publicly funded and private health care ecosystems, regionality (large Canadian urban setting vs United States–wide), as well as differences in the demographic and oncologic characteristics, which together determine the systemic inequities faced by the patient populations in these studies. These factors highlight the challenges of generalizing findings between health care settings. Nonetheless, we believe that the granularity of demographic characterization provided in our study may promote transferability of findings to other tertiary cancer centers located in large, diverse urban centers.

### Future Directions

Some findings of our study may warrant further investigation. First, the majority (83.7%) of VC was provided by phone instead of by video. Here, we did not include a comparison between patients with phone and video visits, as a robust assessment of factors influencing the allocation of VC modalities would also require characterization of provider and disease characteristics. This future analysis is required, as previous work has suggested that marginalized patients participate in video visits less often than nonmarginalized peers due to limitations of technological access and literacy [15]. Second, we found differential access to and use of email and VC by clinic type, echoing findings of other studies [16]. While differential VC use by clinic type may be attributable to clinical reasoning regarding the added benefit of in-person oncologic assessment to VC-amenable biochemical and/or radiographic surveillance in certain clinical contexts, differential access to email and survey responsiveness by clinic type are agnostic to provider preferences and may be indicative of unaccounted intersectional digital divides among subpopulations with lower socioeconomic status [28]. Thus, additional work is required to characterize differences in uptake of email and VC across disease sites, and identify inequitable factors serving as barriers to access and use among specific patient populations. Third, we found that increasing age was associated with greater survey responsiveness and increased satisfaction with several care domains surveyed. This result challenges conventional assumptions about the engagement and satisfaction of older patients with digital health tools. Additional work is required to characterize these age-related differences and tailor custom interventions to improve the patient experience across age groups.

### Limitations

Our study design is dependent on patients having documented postal codes, as well as Statistics Canada’s definitions of deprivation indices. As such, these results, while representative at scale, likely do not reflect the experience of populations who are living on societal margins due to precarious housing. Study of these experiences will require targeted engagement with patients and advocates to gather qualitative and quantitative data about their experiences with email and VC use in the health care setting. Our study has additional limitations to consider. First, patients were not stratified by access to and literacy with technologies required for VC and email, such as personal computers, phones, and high-speed home internet; as a result, our study does not account for the impact of these factors on utilization of and satisfaction with digital health tools in cancer care. Second, although the proportion of individuals without knowledge of English is incorporated in the EC index of the CIMD and language of survey completion was collected, our study’s methodology precluded explicit characterization of the linguistic literacy of nonemailable patients and nonresponders; as such, this may be an unaccounted driver of digital divides. Third, our analysis is not intersectional. Individuals occupying intersecting social identities may have different experiences than members of each individual demographic group they may belong to [29], and thus they may be subject to unique digital divides not captured in our study. Fourth, the adapted patient-reported experience survey used in this study has not been validated. The original survey is a validated instrument [22], and questions added to the survey regarding VC are unlikely to impact its validity [23] (Multimedia Appendix 1; section 1.2, Q3-5); nonetheless, this potential limitation can be noted. Although more work is needed to identify the full scope of digital divides, our study provides encouraging evidence that the rapid systemic adoption of digital health tools during the COVID-19 pandemic equitably maintained access to, use of, and satisfaction with health care participation among numerous demographic indices.

### Conclusions

Our cross-sectional study showed that VC and email are digital health tools that can maintain patient access and experience across patient demographics, which are similar regardless of emailability, digital survey responsiveness, and visit type. Although satisfaction is similar among VC and in-person patients, patients structurally marginalized by ethnocultural, situational, and residential status remain less satisfied with their care. To increase equitable participation in cancer care, digital health tools should be carefully deployed in concert with targeted interventions designed to further characterize the experiences of structurally marginalized patients, proactively identify at-risk patients, and implement practical solutions.

## Appendix

Multimedia Appendix 1 Four dimensions of Canadian Index of Multiple Deprivation, Your Voice Matters survey, and comprehensive study data (Tables S1-S24).

## Abbreviations

CIMD: Canadian Index of Multiple Deprivation
DA: dissemination area
EC: ethnocultural composition
ED: economic dependency
OR: odds ratio
PCCF+: Postal Code Conversion File
RI: residential instability
SMD: standardized mean difference
STROBE: Strengthening the Reporting of Observational Studies in Epidemiology
SV: situational vulnerability
VC: virtual care
