# Supplementary material for: Virtual Care and Electronic Patient Communication During COVID-19: Cross-sectional Study of Inequities Across a Canadian Tertiary Cancer Center
Source: J Med Internet Res. 2022 Nov 4;24(11):e39728. doi: 10.2196/39728 (PMC9640204; doi:10.2196/39728)
Supplement: Multimedia Appendix 1 [file jmir_v24i11e39728_app1.docx]

Supplementary Appendices

[Appendix 1.1 — Four Dimensions of Canadian Index of Multiple Deprivation 3](#_Toc117247767)

[Appendix 1.2 — Your Voice Matters survey 4](#_Toc117247768)

[Appendix 1.3 — Comprehensive Study Data 5](#_Toc117247769)

[*Table S1:* Summary demographics of full cohort, in-person patients, and patients with one or more virtual care visits 6](#_Toc117247770)

[*Table S2:* Summary demographics of full cohort, non-emailable, and emailable patients 7](#_Toc117247771)

[*Table S3:* Summary demographics of emailable patients, survey non-responders, and responders 8](#_Toc117247772)

[*Table S4:* Summary demographics of responders and responders with in-person and virtual care visits 9](#_Toc117247773)

[*Table S5:* Summary of language of respondents 10](#_Toc117247774)

[*Table S6:* Summary of responses to survey question 1 10](#_Toc117247775)

[*Table S7:* Summary of responses to survey question 2 10](#_Toc117247776)

[*Table S8:* Summary of responses to survey question 3 10](#_Toc117247777)

[*Table S9:* Summary of responses to survey questions 4 and 5 11](#_Toc117247778)

[*Table S10:* Summary of responses to survey questions 6-15 11](#_Toc117247779)

[*Table S11:* Clinic types of full cohort, in-person patients, and patients with one or more virtual care visits 13](#_Toc117247780)

[*Table S12:* Clinic types of full cohort, non-emailable, and emailable patients 14](#_Toc117247781)

[*Table S13:* Clinic types of emailable patients, survey non-responders, and responders 15](#_Toc117247782)

[*Table S14:* Clinic types of responders and responders with in-person and virtual care visits 16](#_Toc117247783)

[*Table S15:* Multivariable analyses of association between demographics and satisfaction with listening 17](#_Toc117247784)

[*Table S16:* Multivariable analyses of association between demographics and satisfaction with discussion of physical symptoms 17](#_Toc117247785)

[*Table S17:* Multivariable analyses of association between demographics and satisfaction with discussion of emotional symptoms 17](#_Toc117247786)

[*Table S18:* Multivariable analyses of association between demographics and satisfaction with time spent with patient 17](#_Toc117247787)

[*Table S19:* Multivariable analyses of association between demographics and satisfaction with opportunity to ask questions 18](#_Toc117247788)

[*Table S20:* Multivariable analyses of association between demographics and satisfaction with clear explanations 18](#_Toc117247789)

[*Table S21:* Multivariable analyses of association between demographics and satisfaction with involving patient in decision making 18](#_Toc117247790)

[*Table S22:* Multivariable analyses of association between demographics and satisfaction with care being culturally appropriate 19](#_Toc117247791)

[*Table S23:* Multivariable analyses of association between demographics and satisfaction with respectful treatment 19](#_Toc117247792)

[*Table S24:* Multivariable analyses of association between demographics and satisfaction with overall experience 19](#_Toc117247793)

# Appendix 1.1 — Four Dimensions of Canadian Index of Multiple Deprivation

**Residential Instability**

- Proportion of dwellings that are apartment buildings
- Proportion of dwellings that are owned
- Proportion of persons living alone
- Proportion of the population who moved within the past five years
- Proportion of population that is married or common law*

**Economic dependency**

- Proportion of population aged 65 and older
- Proportion of population participating in labour force (aged 15 and older)*
- Ratio of employment to population*
- Dependency ratio (population aged 0-14 and aged 65 and older divide by population aged 15-64)
- Proportion of population receiving government transfer payments

**Ethno-cultural composition**

- Proportion of population who self-identify as visible minority
- Proportion of population that is foreign-born
- Proportion of population no knowledge of either official language (linguistic isolation)
- Proportion of population who are recent immigrants (arrived in five years prior to Census)

**Situational vulnerability**

- Proportion of population who identifies as Aboriginal
- Proportion of dwellings needing major repair
- Proportion of population aged 25-64 without a high school diploma

*****this indicator is reverse coded opposite of the measure indicated.

Source: Statistics Canada. 2019. “The Canadian Index of Multiple Deprivation”. Statistics Canada Catalogue no. 45-20-0001.

# Appendix 1.2 — Your Voice Matters survey

**Feedback about your visit**

Thank you for taking the time to complete this optional survey. By sharing your thoughts, you are helping improve care for other patients and caregivers.

All information you share will be kept confidential (private) and anonymous (without your name or contact details on it). Your responses will be kept in a secure location. Your treatment will not be affected whether you complete the survey or not.

This survey was made with help from Cancer Care Ontario. It takes less than 5 minutes to complete.

Answer these questions based on your last visit at Princess Margaret Cancer Centre. Rate the items below on a scale of 1 to 5. 5 is the best experience and 1 is the worst experience:

**About you:**

1. You are:

- Filling this survey out for yourself as a patient
- Recording responses for a patient

1. You are a:

- Family member, friend, or caregiver
- Volunteer
- Health care provider
- Other

**Type of visit:**

1. Your visit was:

- In-person (you visited the hospital)
- Virtual (through phone or video call)

1. Were you provided options for the type of visit (in-person, phone, video call)?
2. What type of visit did you have?

- Phone
- OTN video
- MS Teams video call

**Your visit:**

**How would you rate your healthcare provider on the following…**

1. Listened to what you had to say?
2. Discussed any of your physical symptoms?
3. Discussed any of your emotional worries or concerns?
4. Spent enough time with you?
5. Let you ask questions?
6. Explained things in a way you could easily understand?
7. Involved you in decisions (choices) about your care in the way that you wanted?
8. Provided care that you felt was appropriate given your ethnic/cultural background?
9. Treated you with respect?

**Overall:**

1. How would you rate your experience at your last visit

**Thank you for filling out the survey.**

Your Voice Matters!

# Appendix 1.3 — Comprehensive Study Data

BTIPPE: before-tax neighbourhood income per single person equivalent (BTIPPE)

DABTIPPE: neighbourhood income decile before tax distribution

CMA: census metropolitan area

Each choropleth map is scaled to the maximum number of patients in a census tract.

The white circle in each map represents the location of the Princess Margaret Cancer Centre.

## *Table S1:* Summary demographics of full cohort, in-person patients, and patients with one or more virtual care visits

| Covariate | Full cohort (n=42194) | In-person only (n=15759) | ≥ 1 virtual visit  (n=26435) | SMD |
| --- | --- | --- | --- | --- |
| **Age** |  |  |  | 0.072 |
| Mean (sd) | 61.6 (15.5) | 60.9 (15.4) | 62 (15.5) |  |
| Median (IQR) | 64 (52,73) | 63.0 (52.0,72.0) | 64.0 (53.0,73.0) |  |
| **Gender, n (%)** |  |  |  | 0.15 |
| Female | 20388 (48) | 8353 (53) | 12035 (45) |  |
| Male | 21806 (52) | 7406 (47) | 14400 (55) |  |
| **Area, n (%)** |  |  |  | 0.097 |
| Rural area | 2830 (7) | 1210 (7.7) | 1620 (6.1) |  |
| Small population centre (1,000 to 29,999) | 2127 (5) | 921 (5.8) | 1206 (4.6) |  |
| Medium population centre (30,000 to 99,999) | 1494 (4) | 625 (4.0) | 869 (3.3) |  |
| Large urban population centre (100,000 or greater) | 35743 (85) | 13003 (82.5) | 22740 (86.0) |  |
| **BTIPPE x 1000 dollars** |  |  |  | 0.09 |
| Mean (sd) | 62.4 (27.7) | 60.9 (26.1) | 63.3 (28.6) |  |
| Median (IQR) | 58.4 (46.2,72.6) | 57.6 (45.4,70.8) | 58.8 (46.5,73.4) |  |
| **DABTIPPE, n (%)** |  |  |  | 0.088 |
| 1 | 3594 (9) | 1455 (9.2) | 2139 (8.1) |  |
| 2 | 3668 (9) | 1415 (9.0) | 2253 (8.5) |  |
| 3 | 3888 (9) | 1505 (9.6) | 2383 (9.0) |  |
| 4 | 3925 (9) | 1478 (9.4) | 2447 (9.3) |  |
| 5 | 3795 (9) | 1417 (9.0) | 2378 (9.0) |  |
| 6 | 3899 (9) | 1448 (9.2) | 2451 (9.3) |  |
| 7 | 3750 (9) | 1467 (9.3) | 2283 (8.6) |  |
| 8 | 4103 (10) | 1570 (10.0) | 2533 (9.6) |  |
| 9 | 4822 (11) | 1753 (11.1) | 3069 (11.6) |  |
| 10 | 6750 (16) | 2251 (14.3) | 4499 (17.0) |  |
| **Low income, n (%)** |  |  |  | 0.074 |
| No | 39173 (93) | 14816 (94) | 24357 (92) |  |
| Yes | 3021 (7) | 943 (6) | 2078 (8) |  |
| **Residential instability Scores** |  |  |  | 0.055 |
| Mean (sd) | 0.3 (1.2) | 0.3 (1.2) | 0.3 (1.2) |  |
| Median (IQR) | -0.1 (-0.6,1.2) | -0.15 (-0.7,1.1) | -0.1 (-0.6,1.3) |  |
| **Economic dependency Scores** |  |  |  | 0.009 |
| Mean (sd) | -0.1 (1.1) | -0.1 (1.1) | -0.1 (1.1) |  |
| Median (IQR) | -0.2 (-0.8,0.4) | -0.2 (-0.8,0.4) | -0.2 (-0.8,0.4) |  |
| **Ethno cultural composition Scores** |  |  |  | 0.012 |
| Mean (sd) | 0.5 (1.1) | 0.5 (1.1) | 0.5 (1) |  |
| Median (IQR) | 0.3 (-0.4,1.2) | 0.3 (-0.4,1.2) | 0.3 (-0.4,1.2) |  |
| **Situational vulnerability Scores** |  |  |  | 0.069 |
| Mean (sd) | -0.3 (0.8) | -0.3 (0.8) | -0.3 (0.8) |  |
| Median (IQR) | -0.5 (-0.8,0.1) | -0.4 (-0.8,0.1) | -0.5 (-0.8,0) |  |
| **Residential instability Quintile, n (%)** |  |  |  | 0.071 |
| 1 | 6747 (16) | 2529 (16.0) | 4218 (16.0) |  |
| 2 | 7025 (17) | 2835 (18.0) | 4190 (15.9) |  |
| 3 | 7100 (17) | 2732 (17.3) | 4368 (16.5) |  |
| 4 | 7500 (18) | 2720 (17.3) | 4780 (18.1) |  |
| 5 | 13822 (33) | 4943 (31.4) | 8879 (33.6) |  |
| **Economic dependency Quintile, n (%)** |  |  |  | 0.015 |
| 1 | 10764 (26) | 3994 (25.3) | 6770 (25.6) |  |
| 2 | 8685 (21) | 3252 (20.6) | 5433 (20.6) |  |
| 3 | 7983 (19) | 3004 (19.1) | 4979 (18.8) |  |
| 4 | 7398 (18) | 2799 (17.8) | 4599 (17.4) |  |
| 5 | 7364 (17) | 2710 (17.2) | 4654 (17.6) |  |
| **Ethno cultural composition Quintile, n (%)** |  |  |  | 0.081 |
| 1 | 2663 (6) | 1120 ( 7.1) | 1543 ( 5.8) |  |
| 2 | 4616 (11) | 1824 (11.6) | 2792 (10.6) |  |
| 3 | 7606 (18) | 2694 (17.1) | 4912 (18.6) |  |
| 4 | 12195 (29) | 4355 (27.6) | 7840 (29.7) |  |
| 5 | 15114 (36) | 5766 (36.6) | 9348 (35.4) |  |
| **Situational vulnerability Quintile, n (%)** |  |  |  | 0.007 |
| 1 | 14348 (34) | 5053 (32.1) | 9295 (35.2) |  |
| 2 | 9219 (22) | 3477 (22.1) | 5742 (21.7) |  |
| 3 | 7277 (17) | 2773 (17.6) | 4504 (17.0) |  |
| 4 | 6126 (15) | 2396 (15.2) | 3730 (14.1) |  |
| 5 | 5224 (12) | 2060 (13.1) | 3164 (12.0) |  |

## *Table S2:* Summary demographics of full cohort, non-emailable, and emailable patients

| Covariate | Full cohort (n=42194) | Non-emailable (n=29050) | Emailable (n=13144) | SMD |
| --- | --- | --- | --- | --- |
| **Age** |  |  |  | 0.063 |
| Mean (sd) | 61.6 (15.5) | 61.9 (15.6) | 60.9 (15.4) |  |
| Median (IQR) | 64 (52,73) | 64 (53,73) | 63 (52,72) |  |
| **Gender, n (%)** |  |  |  | 0.053 |
| Female | 20388 (48) | 13798 (47) | 6590 (50) |  |
| Male | 21806 (52) | 15252 (53) | 6554 (50) |  |
| **Area, n (%)** |  |  |  | 0.047 |
| Rural area | 2830 (7) | 2042 (7) | 788 (6) |  |
| Small population centre (1,000 to 29,999) | 2127 (5) | 1489 (5) | 638 (5) |  |
| Medium population centre (30,000 to 99,999) | 1494 (4) | 1050 (4) | 444 (3) |  |
| Large urban population centre (100,000 or greater) | 35743 (85) | 24469 (84) | 11274 (86) |  |
| **BTIPPE x 1000 dollars** |  |  |  | 0.045 |
| Mean (sd) | 62.4 (27.7) | 62 (27.7) | 63.3 (27.8) |  |
| Median (IQR) | 58.4 (46.2,72.6) | 58.1 (45.8,72.1) | 59 (46.8,73.4) |  |
| **DABTIPPE, n (%)** |  |  |  | 0.068 |
| 1 | 3594 (9) | 2606 (9) | 988 (8) |  |
| 2 | 3668 (9) | 2549 (9) | 1119 (9) |  |
| 3 | 3888 (9) | 2699 (9) | 1189 (9) |  |
| 4 | 3925 (9) | 2711 (9) | 1214 (9) |  |
| 5 | 3795 (9) | 2579 (9) | 1216 (9) |  |
| 6 | 3899 (9) | 2732 (9) | 1167 (9) |  |
| 7 | 3750 (9) | 2542 (9) | 1208 (9) |  |
| 8 | 4103 (10) | 2834 (10) | 1269 (10) |  |
| 9 | 4822 (11) | 3243 (11) | 1579 (12) |  |
| 10 | 6750 (16) | 4555 (16) | 2195 (17) |  |
| **Low income, n (%)** |  |  |  | 0.01 |
| No | 39173 (93) | 26993 (93) | 12180 (93) |  |
| Yes | 3021 (7) | 2057 (7) | 964 (7) |  |
| **Residential instability Scores** |  |  |  | 0.021 |
| Mean (sd) | 0.3 (1.2) | 0.3 (1.2) | 0.3 (1.2) |  |
| Median (IQR) | -0.1 (-0.6,1.2) | -0.1 (-0.7,1.2) | -0.1 (-0.6,1.2) |  |
| **Economic dependency Scores** |  |  |  | 0.046 |
| Mean (sd) | -0.1 (1.1) | -0.1 (1.1) | -0.1 (1) |  |
| Median (IQR) | -0.2 (-0.8,0.4) | -0.2 (-0.8,0.4) | -0.3 (-0.8,0.3) |  |
| **Ethno cultural composition Scores** |  |  |  | 0.006 |
| Mean (sd) | 0.5 (1.1) | 0.5 (1.1) | 0.5 (1) |  |
| Median (IQR) | 0.3 (-0.4,1.2) | 0.3 (-0.4,1.2) | 0.3 (-0.4,1.2) |  |
| **Situational vulnerability Scores** |  |  |  | 0.078 |
| Mean (sd) | -0.3 (0.8) | -0.3 (0.8) | -0.4 (0.7) |  |
| Median (IQR) | -0.5 (-0.8,0.1) | -0.4 (-0.8,0.1) | -0.5 (-0.8,0) |  |
| **Residential instability Quintile, n (%)** |  |  |  | 0.022 |
| 1 | 6747 (16) | 4680 (16) | 2067 (16) |  |
| 2 | 7025 (17) | 4878 (17) | 2147 (16) |  |
| 3 | 7100 (17) | 4893 (17) | 2207 (17) |  |
| 4 | 7500 (18) | 5165 (18) | 2335 (18) |  |
| 5 | 13822 (33) | 9434 (32) | 4388 (33) |  |
| **Economic dependency Quintile, n (%)** |  |  |  | 0.049 |
| 1 | 10764 (26) | 7238 (25) | 3526 (27) |  |
| 2 | 8685 (21) | 5962 (21) | 2723 (21) |  |
| 3 | 7983 (19) | 5548 (19) | 2435 (19) |  |
| 4 | 7398 (18) | 5192 (18) | 2206 (17) |  |
| 5 | 7364 (17) | 5110 (18) | 2254 (17) |  |
| **Ethno cultural composition Quintile, n (%)** |  |  |  | 0.057 |
| 1 | 2663 (6) | 1928 (7) | 735 (6) |  |
| 2 | 4616 (11) | 3208 (11) | 1408 (11) |  |
| 3 | 7606 (18) | 5147 (18) | 2459 (19) |  |
| 4 | 12195 (29) | 8279 (28) | 3916 (30) |  |
| 5 | 15114 (36) | 10488 (36) | 4626 (35) |  |
| **Situational vulnerability Quintile, n (%)** |  |  |  | 0.082 |
| 1 | 14348 (34) | 9575 (33) | 4773 (36) |  |
| 2 | 9219 (22) | 6400 (22) | 2819 (21) |  |
| 3 | 7277 (17) | 5003 (17) | 2274 (17) |  |
| 4 | 6126 (15) | 4319 (15) | 1807 (14) |  |
| 5 | 5224 (12) | 3753 (13) | 1471 (11) |  |

## *Table S3:* Summary demographics of emailable patients, survey non-responders, and responders

| Covariate | Emailable (n=13144) | Non-responder (n=10274) | Responder (n=2870) | SMD |
| --- | --- | --- | --- | --- |
| **Age** |  |  |  | **0.241** |
| Mean (sd) | 60.9 (15.4) | 60.1 (15.8) | 63.7 (13.4) |  |
| Median (IQR) | 63 (52,72) | 62 (51,71) | 65 (56,73) |  |
| **Gender, n (%)** |  |  |  | 0.066 |
| Female | 6590 (50) | 5077 (49) | 1513 (53) |  |
| Male | 6554 (50) | 5197 (51) | 1357 (47) |  |
| **Area, n (%)** |  |  |  | 0.008 |
| Rural area | 788 (6) | 616 (6) | 172 (6) |  |
| Small population centre (1,000 to 29,999) | 638 (5) | 496 (5) | 142 (5) |  |
| Medium population centre (30,000 to 99,999) | 444 (3) | 345 (3) | 99 (3) |  |
| Large urban population centre (100,000 or greater) | 11274 (86) | 8817 (86) | 2457 (86) |  |
| **BTIPPE x 1000 dollars** |  |  |  | 0.137 |
| Mean (sd) | 63.3 (27.8) | 62.5 (27.4) | 66.3 (29) |  |
| Median (IQR) | 59 (46.8,73.4) | 58.4 (46.3,72.6) | 61.4 (49,76.5) |  |
| **DABTIPPE, n (%)** |  |  |  | 0.182 |
| 1 | 988 (8) | 818 (8) | 170 (6) |  |
| 2 | 1119 (9) | 933 (9) | 186 (6) |  |
| 3 | 1189 (9) | 957 (9) | 232 (8) |  |
| 4 | 1214 (9) | 945 (9) | 269 (9) |  |
| 5 | 1216 (9) | 974 (9) | 242 (8) |  |
| 6 | 1167 (9) | 901 (9) | 266 (9) |  |
| 7 | 1208 (9) | 921 (9) | 287 (10) |  |
| 8 | 1269 (10) | 998 (10) | 271 (9) |  |
| 9 | 1579 (12) | 1221 (12) | 358 (12) |  |
| 10 | 2195 (17) | 1606 (16) | 589 (21) |  |
| **Low income, n (%)** |  |  |  | 0.053 |
| No | 12180 (93) | 9552 (93) | 2628 (92) |  |
| Yes | 964 (7) | 722 (7) | 242 (8) |  |
| **Residential instability Scores** |  |  |  | 0.023 |
| Mean (sd) | 0.3 (1.2) | 0.3 (1.2) | 0.4 (1.2) |  |
| Median (IQR) | -0.1 (-0.6,1.2) | -0.1 (-0.6,1.2) | -0.1 (-0.6,1.3) |  |
| **Economic dependency Scores** |  |  |  | 0.045 |
| Mean (sd) | -0.1 (1) | -0.1 (1) | -0.1 (1.1) |  |
| Median (IQR) | -0.3 (-0.8,0.3) | -0.3 (-0.8,0.3) | -0.3 (-0.8,0.4) |  |
| **Ethno cultural composition Scores** |  |  |  | 0.186 |
| Mean (sd) | 0.5 (1) | 0.5 (1.1) | 0.3 (1) |  |
| Median (IQR) | 0.3 (-0.4,1.2) | 0.3 (-0.4,1.3) | 0.1 (-0.4,0.9) |  |
| **Situational vulnerability Scores** |  |  |  | 0.156 |
| Mean (sd) | -0.4 (0.7) | -0.3 (0.8) | -0.4 (0.7) |  |
| Median (IQR) | -0.5 (-0.8,0) | -0.5 (-0.8,0) | -0.6 (-0.9,-0.1) |  |
| **Residential instability Quintile, n (%)** |  |  |  | 0.046 |
| 1 | 2067 (16) | 1612 (16) | 455 (16) |  |
| 2 | 2147 (16) | 1670 (16) | 477 (17) |  |
| 3 | 2207 (17) | 1750 (17) | 457 (16) |  |
| 4 | 2335 (18) | 1847 (18) | 488 (17) |  |
| 5 | 4388 (33) | 3395 (33) | 993 (35) |  |
| **Economic dependency Quintile, n (%)** |  |  |  | 0.05 |
| 1 | 3526 (27) | 2759 (27) | 767 (27) |  |
| 2 | 2723 (21) | 2151 (21) | 572 (20) |  |
| 3 | 2435 (19) | 1915 (19) | 520 (18) |  |
| 4 | 2206 (17) | 1728 (17) | 478 (17) |  |
| 5 | 2254 (17) | 1721 (17) | 533 (19) |  |
| **Ethno cultural composition Quintile, n (%)** |  |  |  | 0.177 |
| 1 | 735 (6) | 569 (6) | 166 (6) |  |
| 2 | 1408 (11) | 1051 (10) | 357 (12) |  |
| 3 | 2459 (19) | 1854 (18) | 605 (21) |  |
| 4 | 3916 (30) | 3003 (29) | 913 (32) |  |
| 5 | 4626 (35) | 3797 (37) | 829 (29) |  |
| **Situational vulnerability Quintile, n (%)** |  |  |  | 0.15 |
| 1 | 4773 (36) | 3624 (35) | 1149 (40) |  |
| 2 | 2819 (21) | 2161 (21) | 658 (23) |  |
| 3 | 2274 (17) | 1814 (18) | 460 (16) |  |
| 4 | 1807 (14) | 1450 (14) | 357 (12) |  |
| 5 | 1471 (11) | 1225 (12) | 246 (9) |  |

## *Table S4:* Summary demographics of responders and responders with in-person and virtual care visits

69 patients did not provide visit type

| Covariate | Responder (n=2870) | In-person (n=2101) | Virtual (n=700) | SMD |
| --- | --- | --- | --- | --- |
| **Age** |  |  |  | 0.109 |
| Mean (sd) | 63.7 (13.5) | 63.3 (13.7) | 64.8 (13) |  |
| Median (IQR) | 65 (56,73) | 65 (56,73) | 67 (58,74) |  |
| **Gender, n (%)** |  |  |  | 0.197 |
| Female | 1482 (53) | 1163 (55) | 319 (46) |  |
| Male | 1319 (47) | 938 (45) | 381 (54) |  |
| **Area, n (%)** |  |  |  | 0.093 |
| Rural area | 170 (6) | 121 (6) | 49 (7) |  |
| Small population centre (1,000 to 29,999) | 137 (5) | 99 (5) | 38 (5) |  |
| Medium population centre (30,000 to 99,999) | 98 (3) | 67 (3) | 31 (4) |  |
| Large urban population centre (100,000 or greater) | 2396 (86) | 1814 (86) | 582 (83) |  |
| **BTIPPE x 1000 dollars** |  |  |  | 0.116 |
| Mean (sd) | 66.5 (29) | 65.7 (28.3) | 69.1 (30.9) |  |
| Median (IQR) | 61.5 (49.2,76.5) | 61 (48.4,76.5) | 64 (51.5,77.2) |  |
| **DABTIPPE, n (%)** |  |  |  | 0.176 |
| 1 | 158 (6) | 117 (6) | 41 (6) |  |
| 2 | 183 (7) | 147 (7) | 36 (5) |  |
| 3 | 222 (8) | 180 (9) | 42 (6) |  |
| 4 | 261 (9) | 207 (10) | 54 (8) |  |
| 5 | 235 (8) | 179 (9) | 56 (8) |  |
| 6 | 261 (9) | 191 (9) | 70 (10) |  |
| 7 | 283 (10) | 212 (10) | 71 (10) |  |
| 8 | 264 (9) | 197 (9) | 67 (10) |  |
| 9 | 352 (13) | 255 (12) | 97 (14) |  |
| 10 | 582 (21) | 416 (20) | 166 (24) |  |
| **Low income, n (%)** |  |  |  | 0.052 |
| No | 2564 (92) | 1931 (92) | 633 (90) |  |
| Yes | 237 (8) | 170 (8) | 67 (10) |  |
| **Residential instability Scores** |  |  |  | 0.06 |
| Mean (sd) | 0.4 (1.2) | 0.4 (1.2) | 0.3 (1.2) |  |
| Median (IQR) | -0.1 (-0.7,1.3) | 0 (-0.7,1.3) | -0.1 (-0.6,1.2) |  |
| **Economic dependency Scores** |  |  |  | 0.034 |
| Mean (sd) | -0.1 (1.1) | -0.1 (1.1) | -0.1 (1.1) |  |
| Median (IQR) | -0.3 (-0.8,0.4) | -0.3 (-0.8,0.4) | -0.2 (-0.8,0.5) |  |
| **Ethno cultural composition Scores** |  |  |  | 0.18 |
| Mean (sd) | 0.3 (1) | 0.4 (1) | 0.2 (0.9) |  |
| Median (IQR) | 0.1 (-0.5,0.9) | 0.2 (-0.4,1) | 0 (-0.5,0.7) |  |
| **Situational vulnerability Scores** |  |  |  | 0.093 |
| Mean (sd) | -0.5 (0.7) | -0.4 (0.7) | -0.5 (0.7) |  |
| Median (IQR) | -0.6 (-0.9,-0.1) | -0.6 (-0.9,-0.1) | -0.6 (-0.9,-0.2) |  |
| **Residential instability Quintile, n (%)** |  |  |  | 0.097 |
| 1 | 448 (16) | 343 (16) | 105 (15) |  |
| 2 | 464 (17) | 337 (16) | 127 (18) |  |
| 3 | 448 (16) | 326 (16) | 122 (17) |  |
| 4 | 476 (17) | 354 (17) | 122 (17) |  |
| 5 | 965 (34) | 741 (35) | 224 (32) |  |
| **Economic dependency Quintile, n (%)** |  |  |  | 0.1 |
| 1 | 751 (27) | 556 (26) | 195 (28) |  |
| 2 | 561 (20) | 434 (21) | 127 (18) |  |
| 3 | 504 (18) | 375 (18) | 129 (18) |  |
| 4 | 466 (17) | 360 (17) | 106 (15) |  |
| 5 | 519 (19) | 376 (18) | 143 (20) |  |
| **Ethno cultural composition Quintile, n (%)** |  |  |  | 0.183 |
| 1 | 161 (6) | 113 (5) | 48 (7) |  |
| 2 | 354 (13) | 252 (12) | 102 (15) |  |
| 3 | 597 (21) | 429 (20) | 168 (24) |  |
| 4 | 896 (32) | 675 (32) | 221 (32) |  |
| 5 | 793 (28) | 632 (30) | 161 (23) |  |
| **Situational vulnerability Quintile, n (%)** |  |  |  | 0.109 |
| 1 | 1127 (40) | 824 (39) | 303 (43) |  |
| 2 | 641 (23) | 480 (23) | 161 (23) |  |
| 3 | 452 (16) | 344 (16) | 108 (15) |  |
| 4 | 351 (13) | 269 (13) | 82 (12) |  |
| 5 | 230 (8) | 184 (9) | 46 (7) |  |

## *Table S5:* Summary of language of respondents

| Language | n=2870 (100%) |
| --- | --- |
| English | 2811 (98) |
| French | 10 (0) |
| Portuguese | 6 (0) |
| Simplified Chinese | 18 (1) |
| Spanish | 9 (0) |
| Traditional Chinese | 15 (1) |
| Vietnamese | 1 (0) |
| Italian | 0 (0) |

## *Table S6:* Summary of responses to survey question 1

| Patient | n=2870 (100%) |
| --- | --- |
| Other | 294 (11) |
| Patient | 2499 (89) |
| Missing | **77** |

## *Table S7:* Summary of responses to survey question 2

| Role | n=294 (100%) |
| --- | --- |
| Family | 287 (99) |
| Other | 1 (0) |
| Provider | 1 (0) |
| Volunteer | 1 (0) |
| Missing | **4** |

## *Table S8:* Summary of responses to survey question 3

| Visit Type | n=2870 (100%) |
| --- | --- |
| In-person | 2101 (75) |
| Virtual | 700 (25) |
| Missing | **69** |

## *Table S9:* Summary of responses to survey questions 4 and 5

| Provided Options for Visit Type | n (%) |
| --- | --- |
| No | 445 (64) |
| Yes | 251 (36) |
| Missing | 4 |
| **Type of Virtual** |  |
| OTN | 81 (12) |
| Phone | 586 (85) |
| Teams | 26 (4) |
| Missing | 7 |

## *Table S10:* Summary of responses to survey questions 6-15

69 patients did not provide visit type

| Covariate | Full Sample (n=2870) | In-person (n=2101) | Virtual (n=700) |
| --- | --- | --- | --- |
| **Listening, n (%)** |  |  |  |
| 1 | 21 (1) | 16 (1) | 5 (1) |
| 2 | 20 (1) | 15 (1) | 5 (1) |
| 3 | 79 (3) | 57 (3) | 22 (3) |
| 4 | 452 (17) | 350 (17) | 102 (15) |
| 5 | 2152 (79) | 1601 (79) | 551 (80) |
| Missing | 77 | 62 | 15 |
| **Physical Symptoms, n (%)** |  |  |  |
| 1 | 18 (1) | 14 (1) | 4 (1) |
| 2 | 22 (1) | 19 (1) | 3 (0) |
| 3 | 96 (4) | 69 (4) | 27 (4) |
| 4 | 457 (17) | 353 (18) | 104 (16) |
| 5 | 2039 (77) | 1516 (77) | 523 (79) |
| Missing | 169 | 130 | 39 |
| **Emotional Symptoms, n (%)** |  |  |  |
| 1 | 59 (2) | 44 (2) | 15 (3) |
| 2 | 81 (3) | 68 (4) | 13 (2) |
| 3 | 270 (11) | 207 (12) | 63 (11) |
| 4 | 480 (20) | 373 (21) | 107 (19) |
| 5 | 1487 (63) | 1108 (62) | 379 (66) |
| Missing | 424 | 301 | 123 |
| **Enough Time, n (%)** |  |  |  |
| 1 | 33 (1) | 25 (1) | 8 (1) |
| 2 | 56 (2) | 44 (2) | 12 (2) |
| 3 | 155 (6) | 116 (6) | 39 (6) |
| 4 | 530 (20) | 414 (20) | 116 (17) |
| 5 | 1941 (71) | 1434 (71) | 507 (74) |
| Missing | 86 | 68 | 18 |
| **Ask Questions, n (%)** |  |  |  |
| 1 | 19 (1) | 14 (1) | 5 (1) |
| 2 | 26 (1) | 21 (1) | 5 (1) |
| 3 | 101 (4) | 73 (4) | 28 (4) |
| 4 | 457 (17) | 355 (18) | 102 (15) |
| 5 | 2101 (78) | 1559 (77) | 542 (79) |
| Missing | 97 | 79 | 18 |
| **Explain Clearly, n (%)** |  |  |  |
| 1 | 27 (1) | 23 (1) | 4 (1) |
| 2 | 22 (1) | 15 (1) | 7 (1) |
| 3 | 94 (3) | 72 (4) | 22 (3) |
| 4 | 502 (19) | 386 (19) | 116 (17) |
| 5 | 2044 (76) | 1518 (75) | 526 (78) |
| Missing | 112 | 87 | 25 |
| **Involve You, n (%)** |  |  |  |
| 1 | 36 (1) | 28 (1) | 8 (1) |
| 2 | 36 (1) | 25 (1) | 11 (2) |
| 3 | 126 (5) | 101 (5) | 25 (4) |
| 4 | 492 (20) | 374 (20) | 118 (19) |
| 5 | 1797 (72) | 1346 (72) | 451 (74) |
| Missing | 314 | 227 | 87 |
| **Culturally Appropriate, n (%)** |  |  |  |
| 1 | 22 (1) | 16 (1) | 6 (1) |
| 2 | 22 (1) | 18 (1) | 4 (1) |
| 3 | 73 (3) | 56 (3) | 17 (3) |
| 4 | 390 (16) | 296 (16) | 94 (16) |
| 5 | 1905 (79) | 1454 (79) | 451 (79) |
| Missing | 389 | 261 | 128 |
| **Respect, n (%)** |  |  |  |
| 1 | 21 (1) | 18 (1) | 3 (0) |
| 2 | 14 (1) | 10 (0) | 4 (1) |
| 3 | 41 (2) | 33 (2) | 8 (1) |
| 4 | 284 (10) | 207 (10) | 77 (11) |
| 5 | 2360 (87) | 1775 (87) | 585 (86) |
| Missing | 81 | 58 | 23 |
| **Experience Overall, n (%)** |  |  |  |
| 1 | 34 (1) | 23 (1) | 11 (2) |
| 2 | 34 (1) | 19 (1) | 15 (2) |
| 3 | 122 (4) | 90 (4) | 32 (5) |
| 4 | 702 (25) | 537 (26) | 165 (24) |
| 5 | 1870 (68) | 1411 (68) | 459 (67) |

##

## *Table S11:* Clinic types of full cohort, in-person patients, and patients with one or more virtual care visits

| **Clinic type, n (%)** | **Full cohort (n=42194)** | **In-person only (n=15759)** | **≥ 1 virtual visit**  **(n=26435)** | **SMD** |
| --- | --- | --- | --- | --- |
| ADOLESCENT & YOUNG ADULT PROGRAM | 1 ( 0.0) | 0 ( 0.0) | 1 ( 0.0) | 1.112 |
| ADOPTIVE CELL THERAPY | 1 ( 0.0) | 0 ( 0.0) | 1 ( 0.0) |  |
| ADULT RADIATION LATE EFFECTS | 87 ( 0.2) | 27 ( 0.2) | 60 ( 0.2) |  |
| BONE MARROW TRANSPLANT | 837 ( 2.0) | 551 ( 3.5) | 286 ( 1.1) |  |
| BRAIN METASTASIS | 187 ( 0.4) | 120 ( 0.8) | 67 ( 0.3) |  |
| BREAST | 3478 ( 8.2) | 762 ( 4.8) | 2716 (10.3) |  |
| CENTRAL NERVOUS SYSTEM | 1071 ( 2.5) | 286 ( 1.8) | 785 ( 3.0) |  |
| ENDOCRINE | 1601 ( 3.8) | 919 ( 5.8) | 682 ( 2.6) |  |
| ENT | 4594 (10.9) | 2875 (18.2) | 1719 ( 6.5) |  |
| EYE | 1982 ( 4.7) | 1866 (11.8) | 116 ( 0.4) |  |
| FAMILIAL CANCER CLINIC | 91 ( 0.2) | 5 ( 0.0) | 86 ( 0.3) |  |
| GASTROINTESTINAL | 4220 (10.0) | 1302 ( 8.3) | 2918 (11.0) |  |
| GENITOURINARY | 8885 (21.1) | 1099 ( 7.0) | 7786 (29.5) |  |
| TESTES | 742 ( 1.8) | 82 ( 0.5) | 660 ( 2.5) |  |
| GYNECOLOGIC | 3316 ( 7.9) | 1707 (10.8) | 1609 ( 6.1) |  |
| HIGH RISK / FAMILIAL BREAST CANCER | 45 ( 0.1) | 1 ( 0.0) | 44 ( 0.2) |  |
| LEUKEMIA | 1602 ( 3.8) | 1026 ( 6.5) | 576 ( 2.2) |  |
| LYMPHOMA | 2286 ( 5.4) | 1086 ( 6.9) | 1200 ( 4.5) |  |
| MELANOMA | 830 ( 2.0) | 254 ( 1.6) | 576 ( 2.2) |  |
| MYELOMA | 1067 ( 2.5) | 405 ( 2.6) | 662 ( 2.5) |  |
| OLDER ADULTS WITH CANCER CLINIC | 2 ( 0.0) | 0 ( 0.0) | 2 ( 0.0) |  |
| PAIN | 49 ( 0.1) | 1 ( 0.0) | 48 ( 0.2) |  |
| PALLIATIVE ONCOLOGY | 265 ( 0.6) | 5 ( 0.0) | 260 ( 1.0) |  |
| PALLIATIVE RADIATION ONCOLOGY | 88 ( 0.2) | 63 ( 0.4) | 25 ( 0.1) |  |
| PEDIATRIC ONCOLOGY | 241 ( 0.6) | 3 ( 0.0) | 238 ( 0.9) |  |
| PERITONEAL MALIGNANCY | 29 ( 0.1) | 1 ( 0.0) | 28 ( 0.1) |  |
| PHASE 1 ONCOLOGY | 1 ( 0.0) | 0 ( 0.0) | 1 ( 0.0) |  |
| PSYCHO-SOCIAL ONCOLOGY | 617 ( 1.5) | 23 ( 0.1) | 594 ( 2.2) |  |
| RADIATION THERAPY REVIEW | 2 ( 0.0) | 1 ( 0.0) | 1 ( 0.0) |  |
| RAPID DIAGNOSTIC CLINIC | 610 ( 1.4) | 106 ( 0.7) | 504 ( 1.9) |  |
| SARCOMA | 829 ( 2.0) | 432 ( 2.7) | 397 ( 1.5) |  |
| SURVIVORSHIP CLINIC | 113 ( 0.3) | 11 ( 0.1) | 102 ( 0.4) |  |
| TESTING CLINIC | 1 ( 0.0) | 0 ( 0.0) | 1 ( 0.0) |  |
| THORACIC | 2196 ( 5.2) | 661 ( 4.2) | 1535 ( 5.8) |  |
| UNKNOWN PRIMARY TUMOUR CLINIC | 10 ( 0.0) | 2 ( 0.0) | 8 ( 0.0) |  |
| UROLOGY CLINIC - OFF SITE | 135 ( 0.3) | 12 ( 0.1) | 123 ( 0.5) |  |
| Not specified | 83 ( 0.2) | 65 ( 0.4) | 18 ( 0.1) |  |

## *Table S12:* Clinic types of full cohort, non-emailable, and emailable patients

| **Clinic type, n (%)** | **Full cohort (n=42194)** | **Non-emailable (n=29050)** | **Emailable (n=13144)** | **SMD** |
| --- | --- | --- | --- | --- |
| ADOLESCENT & YOUNG ADULT PROGRAM | 1 ( 0.0) | 0 ( 0.0) | 1 ( 0.0) | 0.612 |
| ADOPTIVE CELL THERAPY | 1 ( 0.0) | 0 ( 0.0) | 1 ( 0.0) |  |
| ADULT RADIATION LATE EFFECTS | 87 ( 0.2) | 61 ( 0.2) | 26 ( 0.2) |  |
| BONE MARROW TRANSPLANT | 837 ( 2.0) | 424 ( 1.5) | 413 ( 3.1) |  |
| BRAIN METASTASIS | 187 ( 0.4) | 105 ( 0.4) | 82 ( 0.6) |  |
| BREAST | 3478 ( 8.2) | 2044 ( 7.0) | 1434 (10.9) |  |
| CENTRAL NERVOUS SYSTEM | 1071 ( 2.5) | 730 ( 2.5) | 341 ( 2.6) |  |
| ENDOCRINE | 1601 ( 3.8) | 1405 ( 4.8) | 196 ( 1.5) |  |
| ENT | 4594 (10.9) | 3800 (13.1) | 794 ( 6.0) |  |
| EYE | 1982 ( 4.7) | 1792 ( 6.2) | 190 ( 1.4) |  |
| FAMILIAL CANCER CLINIC | 91 ( 0.2) | 52 ( 0.2) | 39 ( 0.3) |  |
| GASTROINTESTINAL | 4220 (10.0) | 2768 ( 9.5) | 1452 (11.0) |  |
| GENITOURINARY | 8885 (21.1) | 6699 (23.1) | 2186 (16.6) |  |
| TESTES | 742 ( 1.8) | 439 ( 1.5) | 303 ( 2.3) |  |
| GYNECOLOGIC | 3316 ( 7.9) | 1926 ( 6.6) | 1390 (10.6) |  |
| HIGH RISK / FAMILIAL BREAST CANCER | 45 ( 0.1) | 35 ( 0.1) | 10 ( 0.1) |  |
| LEUKEMIA | 1602 ( 3.8) | 1086 ( 3.7) | 516 ( 3.9) |  |
| LYMPHOMA | 2286 ( 5.4) | 1283 ( 4.4) | 1003 ( 7.6) |  |
| MELANOMA | 830 ( 2.0) | 338 ( 1.2) | 492 ( 3.7) |  |
| MYELOMA | 1067 ( 2.5) | 532 ( 1.8) | 535 ( 4.1) |  |
| OLDER ADULTS WITH CANCER CLINIC | 2 ( 0.0) | 2 ( 0.0) | 0 ( 0.0) |  |
| PAIN | 49 ( 0.1) | 27 ( 0.1) | 22 ( 0.2) |  |
| PALLIATIVE ONCOLOGY | 265 ( 0.6) | 36 ( 0.1) | 229 ( 1.7) |  |
| PALLIATIVE RADIATION ONCOLOGY | 88 ( 0.2) | 51 ( 0.2) | 37 ( 0.3) |  |
| PEDIATRIC ONCOLOGY | 241 ( 0.6) | 200 ( 0.7) | 41 ( 0.3) |  |
| PERITONEAL MALIGNANCY | 29 ( 0.1) | 26 ( 0.1) | 3 ( 0.0) |  |
| PHASE 1 ONCOLOGY | 1 ( 0.0) | 0 ( 0.0) | 1 ( 0.0) |  |
| PSYCHO-SOCIAL ONCOLOGY | 617 ( 1.5) | 389 ( 1.3) | 228 ( 1.7) |  |
| RADIATION THERAPY REVIEW | 2 ( 0.0) | 2 ( 0.0) | 0 ( 0.0) |  |
| RAPID DIAGNOSTIC CLINIC | 610 ( 1.4) | 559 ( 1.9) | 51 ( 0.4) |  |
| SARCOMA | 829 ( 2.0) | 593 ( 2.0) | 236 ( 1.8) |  |
| SURVIVORSHIP CLINIC | 113 ( 0.3) | 79 ( 0.3) | 34 ( 0.3) |  |
| TESTING CLINIC | 1 ( 0.0) | 1 ( 0.0) | 0 ( 0.0) |  |
| THORACIC | 2196 ( 5.2) | 1399 ( 4.8) | 797 ( 6.1) |  |
| UNKNOWN PRIMARY TUMOUR CLINIC | 10 ( 0.0) | 2 ( 0.0) | 8 ( 0.1) |  |
| UROLOGY CLINIC - OFF SITE | 135 ( 0.3) | 108 ( 0.4) | 27 ( 0.2) |  |
| Not specified | 83 ( 0.2) | 57 ( 0.2) | 26 ( 0.2) |  |

## *Table S13:* Clinic types of emailable patients, survey non-responders, and responders

| **Clinic type, n(%)** | **Emailable (n=13144)** | **Non-responder (n=10274)** | **Responder (n=2870)** | **SMD** |
| --- | --- | --- | --- | --- |
| ADOLESCENT & YOUNG ADULT PROGRAM | 1 ( 0.0) | 0 ( 0.0) | 1 ( 0.0) | 0.247 |
| ADOPTIVE CELL THERAPY | 1 ( 0.0) | 1 ( 0.0) | 0 ( 0.0) |  |
| ADULT RADIATION LATE EFFECTS | 26 ( 0.2) | 19 ( 0.2) | 7 ( 0.2) |  |
| BONE MARROW TRANSPLANT | 413 ( 3.1) | 311 ( 3.0) | 102 ( 3.6) |  |
| BRAIN METASTASIS | 82 ( 0.6) | 68 ( 0.7) | 14 ( 0.5) |  |
| BREAST | 1434 (10.9) | 1085 (10.6) | 349 (12.2) |  |
| CENTRAL NERVOUS SYSTEM | 341 ( 2.6) | 286 ( 2.8) | 55 ( 1.9) |  |
| ENDOCRINE | 196 ( 1.5) | 170 ( 1.7) | 26 ( 0.9) |  |
| ENT | 794 ( 6.0) | 621 ( 6.0) | 173 ( 6.0) |  |
| EYE | 190 ( 1.4) | 149 ( 1.5) | 41 ( 1.4) |  |
| FAMILIAL CANCER CLINIC | 39 ( 0.3) | 31 ( 0.3) | 8 ( 0.3) |  |
| GASTROINTESTINAL | 1452 (11.0) | 1152 (11.2) | 300 (10.5) |  |
| GENITOURINARY | 2186 (16.6) | 1753 (17.1) | 433 (15.1) |  |
| TESTES | 303 ( 2.3) | 267 ( 2.6) | 36 ( 1.3) |  |
| GYNECOLOGIC | 1390 (10.6) | 1035 (10.1) | 355 (12.4) |  |
| HIGH RISK / FAMILIAL BREAST CANCER | 10 ( 0.1) | 8 ( 0.1) | 2 ( 0.1) |  |
| LEUKEMIA | 516 ( 3.9) | 411 ( 4.0) | 105 ( 3.7) |  |
| LYMPHOMA | 1003 ( 7.6) | 766 ( 7.5) | 237 ( 8.3) |  |
| MELANOMA | 492 ( 3.7) | 340 ( 3.3) | 152 ( 5.3) |  |
| MYELOMA | 535 ( 4.1) | 388 ( 3.8) | 147 ( 5.1) |  |
| PAIN | 22 ( 0.2) | 21 ( 0.2) | 1 ( 0.0) |  |
| PALLIATIVE ONCOLOGY | 229 ( 1.7) | 187 ( 1.8) | 42 ( 1.5) |  |
| PALLIATIVE RADIATION ONCOLOGY PRG | 37 ( 0.3) | 32 ( 0.3) | 5 ( 0.2) |  |
| PEDIATRIC ONCOLOGY | 41 ( 0.3) | 34 ( 0.3) | 7 ( 0.2) |  |
| PERITONEAL MALIGNANCY ONCOLOGY | 3 ( 0.0) | 3 ( 0.0) | 0 ( 0.0) |  |
| PHASE 1 ONCOLOGY | 1 ( 0.0) | 0 ( 0.0) | 1 ( 0.0) |  |
| PSYCHO-SOCIAL ONCOLOGY | 228 ( 1.7) | 179 ( 1.7) | 49 ( 1.7) |  |
| RAPID DIAGNOSTIC CLINIC | 51 ( 0.4) | 47 ( 0.5) | 4 ( 0.1) |  |
| SARCOMA | 236 ( 1.8) | 196 ( 1.9) | 40 ( 1.4) |  |
| SURVIVORSHIP CLINIC | 34 ( 0.3) | 32 ( 0.3) | 2 ( 0.1) |  |
| THORACIC | 797 ( 6.1) | 627 ( 6.1) | 170 ( 5.9) |  |
| UNKNOWN PRIMARY TUMOUR CLINIC | 8 ( 0.1) | 7 ( 0.1) | 1 ( 0.0) |  |
| UROLOGY CLINIC - OFF SITE | 27 ( 0.2) | 24 ( 0.2) | 3 ( 0.1) |  |
| Not specified | 26 ( 0.2) | 24 ( 0.2) | 2 ( 0.1) |  |

## *Table S14:* Clinic types of responders and responders with in-person and virtual care visits

69 patients did not provide visit type

| **Clinic type, n (%)** | **Responder (n=2870)** | **In-person (n=2101)** | **Virtual (n=700)** | **SMD** |
| --- | --- | --- | --- | --- |
| ADOLESCENT & YOUNG ADULT PROGRAM | 1 ( 0.0) | 1 ( 0.0) | 0 ( 0.0) | 0.646 |
| ADULT RADIATION LATE EFFECTS | 7 ( 0.2) | 5 ( 0.2) | 2 ( 0.3) |  |
| BONE MARROW TRANSPLANT | 102 ( 3.6) | 85 ( 4.0) | 16 ( 2.3) |  |
| BRAIN METASTASIS | 14 ( 0.5) | 9 ( 0.4) | 3 ( 0.4) |  |
| BREAST | 349 (12.2) | 261 (12.4) | 83 (11.9) |  |
| CENTRAL NERVOUS SYSTEM | 55 ( 1.9) | 41 ( 2.0) | 12 ( 1.7) |  |
| ENDOCRINE | 26 ( 0.9) | 21 ( 1.0) | 3 ( 0.4) |  |
| ENT | 173 ( 6.0) | 151 ( 7.2) | 17 ( 2.4) |  |
| EYE | 41 ( 1.4) | 36 ( 1.7) | 5 ( 0.7) |  |
| FAMILIAL CANCER CLINIC | 8 ( 0.3) | 6 ( 0.3) | 2 ( 0.3) |  |
| GASTROINTESTINAL | 300 (10.5) | 238 (11.3) | 50 ( 7.1) |  |
| GENITOURINARY | 433 (15.1) | 219 (10.4) | 204 (29.1) |  |
| TESTES | 36 ( 1.3) | 26 ( 1.2) | 10 ( 1.4) |  |
| GYNECOLOGIC | 355 (12.4) | 288 (13.7) | 61 ( 8.7) |  |
| HIGH RISK / FAMILIAL BREAST CANCER | 2 ( 0.1) | 2 ( 0.1) | 0 ( 0.0) |  |
| LEUKEMIA | 105 ( 3.7) | 79 ( 3.8) | 23 ( 3.3) |  |
| LYMPHOMA | 237 ( 8.3) | 169 ( 8.0) | 66 ( 9.4) |  |
| MELANOMA | 152 ( 5.3) | 121 ( 5.8) | 28 ( 4.0) |  |
| MYELOMA | 147 ( 5.1) | 98 ( 4.7) | 41 ( 5.9) |  |
| PAIN | 1 ( 0.0) | 1 ( 0.0) | 0 ( 0.0) |  |
| PALLIATIVE ONCOLOGY | 42 ( 1.5) | 27 ( 1.3) | 14 ( 2.0) |  |
| PALLIATIVE RADIATION ONCOLOGY PRG | 5 ( 0.2) | 5 ( 0.2) | 0 ( 0.0) |  |
| PEDIATRIC ONCOLOGY | 7 ( 0.2) | 0 ( 0.0) | 7 ( 1.0) |  |
| PHASE 1 ONCOLOGY | 1 ( 0.0) | 1 ( 0.0) | 0 ( 0.0) |  |
| PSYCHO-SOCIAL ONCOLOGY | 49 ( 1.7) | 31 ( 1.5) | 18 ( 2.6) |  |
| RAPID DIAGNOSTIC CLINIC | 4 ( 0.1) | 4 ( 0.2) | 0 ( 0.0) |  |
| SARCOMA | 40 ( 1.4) | 36 ( 1.7) | 4 ( 0.6) |  |
| SURVIVORSHIP CLINIC | 2 ( 0.1) | 1 ( 0.0) | 1 ( 0.1) |  |
| THORACIC | 170 ( 5.9) | 137 ( 6.5) | 26 ( 3.7) |  |
| UNKNOWN PRIMARY TUMOUR CLINIC | 1 ( 0.0) | 0 ( 0.0) | 1 ( 0.1) |  |
| UROLOGY CLINIC - OFF SITE | 3 ( 0.1) | 1 ( 0.0) | 2 ( 0.3) |  |
| Not specified | 2 ( 0.1) | 1 ( 0.0) | 1 ( 0.1) |  |

## *Table S15:* Multivariable analyses of association between demographics and satisfaction with listening

Based on responses to survey question 6

| Covariate | OR (95%CI) | p-value |
| --- | --- | --- |
| **Age per 10 years** | 1.22 (1.07,1.39) | **0.0035** |
| **Gender** |  | **0.014** |
| Female | reference |  |
| Male | 1.64 (1.11,2.44) |  |
| **BTIPPE (log scale)** | 0.9 (0.39,2.12) | 0.82 |
| **Residential instability Scores** | 0.9 (0.74,1.1) | 0.3 |
| **Economic dependency Scores** | 1.16 (0.95,1.42) | 0.15 |
| **Ethno cultural composition Scores** | 0.98 (0.79,1.21) | 0.82 |
| **Situational vulnerability Scores** | 0.85 (0.59,1.22) | 0.37 |
| **Visit Type** |  | 0.4 |
| In-person | reference |  |
| Virtual | 0.83 (0.55,1.27) |  |

## *Table S16:* Multivariable analyses of association between demographics and satisfaction with discussion of physical symptoms

Based on responses to survey question 7

| Covariate | OR (95%CI) | p-value |
| --- | --- | --- |
| **Age per 10 years** | 1.07 (0.94,1.21) | 0.34 |
| **Gender** |  | 0.14 |
| Female | reference |  |
| Male | 1.31 (0.91,1.88) |  |
| **BTIPPE (log scale)** | 0.7 (0.32,1.54) | 0.37 |
| **Residential instability Scores** | 0.81 (0.68,0.97) | **0.024** |
| **Economic dependency Scores** | 1.17 (0.97,1.41) | 0.11 |
| **Ethno cultural composition Scores** | 0.99 (0.81,1.21) | 0.91 |
| **Situational vulnerability Scores** | 0.67 (0.48,0.93) | **0.016** |
| **Visit Type** |  | 0.72 |
| In-person | reference |  |
| Virtual | 0.93 (0.62,1.39) |  |

## *Table S17:* Multivariable analyses of association between demographics and satisfaction with discussion of emotional symptoms

Based on responses to survey question 8

| Covariate | OR (95%CI) | p-value |
| --- | --- | --- |
| **Age per 10 years** | 1.08 (0.99,1.17) | 0.069 |
| **Gender** |  | 0.35 |
| Female | reference |  |
| Male | 1.11 (0.89,1.38) |  |
| **BTIPPE (log scale)** | 0.93 (0.57,1.51) | 0.77 |
| **Residential instability Scores** | 0.86 (0.77,0.96) | **0.0092** |
| **Economic dependency Scores** | 1.01 (0.92,1.12) | 0.78 |
| **Ethno cultural composition Scores** | 1.04 (0.92,1.18) | 0.52 |
| **Situational vulnerability Scores** | 1.01 (0.81,1.24) | 0.96 |
| **Visit Type** |  | 0.37 |
| In-person | reference |  |
| Virtual | 1.12 (0.87,1.45) |  |

## *Table S18:* Multivariable analyses of association between demographics and satisfaction with time spent with patient

Based on responses to survey question 9

| Covariate | OR (95%CI) | p-value |
| --- | --- | --- |
| **Age per 10 years** | 1.23 (1.12,1.35) | $<$**0.001** |
| **Gender** |  | 0.53 |
| Female | reference |  |
| Male | 1.09 (0.83,1.43) |  |
| **BTIPPE (log scale)** | 0.92 (0.51,1.69) | 0.8 |
| **Residential instability Scores** | 0.85 (0.74,0.98) | **0.024** |
| **Economic dependency Scores** | 1.01 (0.89,1.15) | 0.84 |
| **Ethno cultural composition Scores** | 1.01 (0.87,1.18) | 0.86 |
| **Situational vulnerability Scores** | 0.88 (0.68,1.14) | 0.32 |
| **Visit Type** |  | 0.98 |
| In-person | reference |  |
| Virtual | 1 (0.73,1.36) |  |

## *Table S19:* Multivariable analyses of association between demographics and satisfaction with opportunity to ask questions

Based on responses to survey question 10

| Covariate | OR (95%CI) | p-value |
| --- | --- | --- |
| **Age per 10 years** | 1.22 (1.08,1.38) | **0.0011** |
| **Gender** |  | 0.2 |
| Female | reference |  |
| Male | 1.25 (0.88,1.78) |  |
| **BTIPPE (log scale)** | 0.85 (0.4,1.82) | 0.68 |
| **Residential instability Scores** | 0.9 (0.75,1.08) | 0.25 |
| **Economic dependency Scores** | 1.05 (0.88,1.25) | 0.56 |
| **Ethno cultural composition Scores** | 0.94 (0.78,1.14) | 0.54 |
| **Situational vulnerability Scores** | 0.8 (0.58,1.1) | 0.17 |
| **Visit Type** |  | 0.51 |
| In-person | reference |  |
| Virtual | 0.88 (0.6,1.29) |  |

## *Table S20:* Multivariable analyses of association between demographics and satisfaction with clear explanations

Based on responses to survey question 11

| Covariate | OR (95%CI) | p-value |
| --- | --- | --- |
| **Age per 10 years** | 1.17 (1.04,1.33) | **0.01** |
| **Gender** |  | 0.83 |
| Female | reference |  |
| Male | 1.04 (0.73,1.47) |  |
| **BTIPPE (log scale)** | 0.82 (0.38,1.76) | 0.61 |
| **Residential instability Scores** | 0.87 (0.73,1.04) | 0.14 |
| **Economic dependency Scores** | 1.09 (0.91,1.3) | 0.33 |
| **Ethno cultural composition Scores** | 1.05 (0.86,1.28) | 0.61 |
| **Situational vulnerability Scores** | 0.76 (0.55,1.06) | 0.1 |
| **Visit Type** |  | 0.74 |
| In-person | reference |  |
| Virtual | 1.07 (0.72,1.6) |  |

## *Table S21:* Multivariable analyses of association between demographics and satisfaction with involving patient in decision making

Based on responses to survey question 12

| Covariate | OR (95%CI) | p-value |
| --- | --- | --- |
| **Age per 10 years** | 1.17 (1.05,1.3) | **0.0035** |
| **Gender** |  | 0.27 |
| Female | reference |  |
| Male | 1.19 (0.88,1.6) |  |
| **BTIPPE (log scale)** | 0.69 (0.36,1.32) | 0.27 |
| **Residential instability Scores** | 0.91 (0.78,1.06) | 0.24 |
| **Economic dependency Scores** | 1 (0.87,1.16) | 0.96 |
| **Ethno cultural composition Scores** | 0.94 (0.8,1.11) | 0.45 |
| **Situational vulnerability Scores** | 0.76 (0.58,1) | 0.052 |
| **Visit Type** |  | 0.65 |
| In-person | reference |  |
| Virtual | 1.09 (0.76,1.54) |  |

## *Table S22:* Multivariable analyses of association between demographics and satisfaction with care being culturally appropriate

Based on responses to survey question 13

| Covariate | OR (95%CI) | p-value |
| --- | --- | --- |
| **Age per 10 years** | 1.11 (0.97,1.28) | 0.12 |
| **Gender** |  | 0.77 |
| Female | reference |  |
| Male | 1.06 (0.72,1.55) |  |
| **BTIPPE (log scale)** | 0.74 (0.33,1.7) | 0.49 |
| **Residential instability Scores** | 1.02 (0.83,1.25) | 0.83 |
| **Economic dependency Scores** | 0.94 (0.79,1.13) | 0.5 |
| **Ethno cultural composition Scores** | 0.7 (0.57,0.86) | $<$**0.001** |
| **Situational vulnerability Scores** | 1.04 (0.72,1.5) | 0.84 |
| **Visit Type** |  | 0.88 |
| In-person | reference |  |
| Virtual | 0.97 (0.62,1.51) |  |

## *Table S23:* Multivariable analyses of association between demographics and satisfaction with respectful treatment

Based on responses to survey question 14

| Covariate | OR (95%CI) | p-value |
| --- | --- | --- |
| **Age per 10 years** | 1.3 (1.11,1.53) | **0.0011** |
| **Gender** |  | 0.24 |
| Female | reference |  |
| Male | 1.34 (0.83,2.16) |  |
| **BTIPPE (log scale)** | 0.66 (0.24,1.83) | 0.42 |
| **Residential instability Scores** | 0.94 (0.74,1.2) | 0.62 |
| **Economic dependency Scores** | 1.19 (0.91,1.56) | 0.2 |
| **Ethno cultural composition Scores** | 0.92 (0.71,1.2) | 0.54 |
| **Situational vulnerability Scores** | 0.73 (0.47,1.14) | 0.17 |
| **Visit Type** |  | 0.48 |
| In-person | reference |  |
| Virtual | 1.23 (0.69,2.2) |  |

## *Table S24:* Multivariable analyses of association between demographics and satisfaction with overall experience

Based on responses to survey question 15

| Covariate | OR (95%CI) | p-value |
| --- | --- | --- |
| **Age per 10 years** | 1.28 (1.16,1.43) | $<$**0.001** |
| **Gender** |  | 0.28 |
| Female | reference |  |
| Male | 1.18 (0.87,1.61) |  |
| **BTIPPE (log scale)** | 0.67 (0.35,1.28) | 0.22 |
| **Residential instability Scores** | 0.89 (0.76,1.04) | 0.15 |
| **Economic dependency Scores** | 1.15 (0.98,1.35) | 0.093 |
| **Ethno cultural composition Scores** | 1 (0.84,1.19) | 0.98 |
| **Situational vulnerability Scores** | 0.8 (0.6,1.08) | 0.14 |
| **Visit Type** |  | **0.02** |
| In-person | reference |  |
| Virtual | 0.68 (0.49,0.94) |  |
